# Dissection of the interventricular septum: Echocardiographic features

**DOI:** 10.1097/MD.0000000000006191

**Published:** 2017-03-10

**Authors:** Xiaoyan Gu, Yihua He, Shurong Luan, Ying Zhao, Lin Sun, Hongjia Zhang, J.V. Ian Nixon

**Affiliations:** a Department of Ultrasound, Beijing Anzhen Hospital, Capital Medical University, Beijing, P.R. China; b The Pauley Heart Center, Virginal Commonwealth University School of Medicine, Richmond, VA, USA.

**Keywords:** dissection of the interventricular septum, sinus of Valsalva aneurysms, transthoracic echocardiography

## Abstract

Dissection of the interventricular septum (IVS) is an extremely rare entity. An institutional echocardiographic database was retrospectively reviewed; 13 patients with a diagnosis of IVS dissection were found and confirmed by cardiac surgery. The purposes of the study were: to determine the value of transthoracic echocardiography (TTE) in establishing the diagnosis of IVS dissection, and to detail the TTE features of IVS dissection.

Thirteen patients with IVS dissection diagnosed by TTE, 8 males and 5 females were taken from 789,114 TTE studies performed between 1985 and 2014. All underwent cardiac surgery during which their diagnosis was confirmed. The etiology, location, 2-dimensional morphology, and color Doppler findings of IVS dissection were noted.

The right sinus of Valsalva (SOV) was involved in 11 of the 13 patients. In 5 patients, a single aneurysm of the right SOV was seen dissecting into the IVS. One patient with a combination of a bicuspid aortic valve and a right SOV aneurysm dissected into the IVS. In 4 patients, aortic valve infective endocarditis resulted in IVS dissection. In 1 patient, mechanical aortic valve prosthetic replacement was complicated by annular detachment and a severe paravalvular leak causing IVS dissection. In all 11 patients, TTE showed a dissecting cystic-like mass in the IVS from the base to the mid-septum or confined to the septal base. The path of the dissection in these 11 patients was traced to the right SOV and communications between the IVS dissection and the aortic root were identified. In the remaining 2 patients, IVS dissection followed septal rupture due to a myocardial infarction, and communication was seen between the IVS dissection and the right ventricle.

The study showed that most of the dissections of the IVS commence in the right SOV, due to either congenital anomalies or infective endocarditis, or following aortic valve replacement or myocardial infarction. The TTE characteristic of IVS dissection is a cystic-like mass seen in the IVS.

## 1 Introduction

Interventricular septal dissection is a rare anomaly of the interventricular septum (IVS). It may be result from an aneurysm of one of the sinuses of Valsalva (SOV), bacterial endocarditis, trauma, cardiac surgery, endomyocardial biopsy, or a congenital myocardial developmental anomaly.^[1–5]^ IVS dissection may cause significant anatomical and hemodynamic derangements. It has a progressive course and a poor prognosis.^[6]^ It may cause arrhythmias, conduction abnormalities, or congestive heart failure. Few cases have been reported. An institutional echocardiographic database review identified 13 patients with the diagnosis of IVS dissection confirmed by cardiac surgery. The purpose of our study was to determine the value of transthoracic echocardiography (TTE) in establishing the diagnosis of dissection of the IVS, and to summarize the TTE features of IVS dissection.

## 2 Materials and methods

### 2.1 Patients

The time period of the retrospective review was from January 1985 to January 2014. During this time, 789,114 TTE studies performed by a cardiologist at the Beijing Anzhen Hospital, Beijing, China. This study's protocol was implemented with approval from the review board of Beijing Anzhen Hospital. Thirteen patients with IVS dissection diagnosed by TTE and confirmed by cardiac surgery, 8 males and 5 females (age range: 36–75 years; mean: 52 ± 12 years).

### 2.2 Echocardiographic equipment and study protocols

The echocardiographic findings were summarized. The etiology, location, 2-dimensional morphology, and color Doppler findings of the IVS dissection were noted. Clinical data were obtained from medical and pathologic records, including age, symptoms on presentation, and medical history. The study protocol was approved by the review board of Beijing Anzhen Hospital. TTE examinations were performed on each of the patients when indicated by their cardiac symptoms using either GE Healthcare (Milwaukee, WI) or Philips Medical Systems (Andover, MA) echocardiographic equipment. A standard echocardiographic exam was performed according to ASE guidelines. The parasternal long axis view, parasternal short-axis view, apical 4 chamber view, apical 5 chamber view, apical long axis view, and subcostal view were utilized.

## 3 Results

From 1985 to 2014, 789,114 transthoracic echocardiographic studies were reviewed. The clinical data, echocardiographic findings, and pathological details of the 13 patients are listed in Table 1.

**Table 1:**
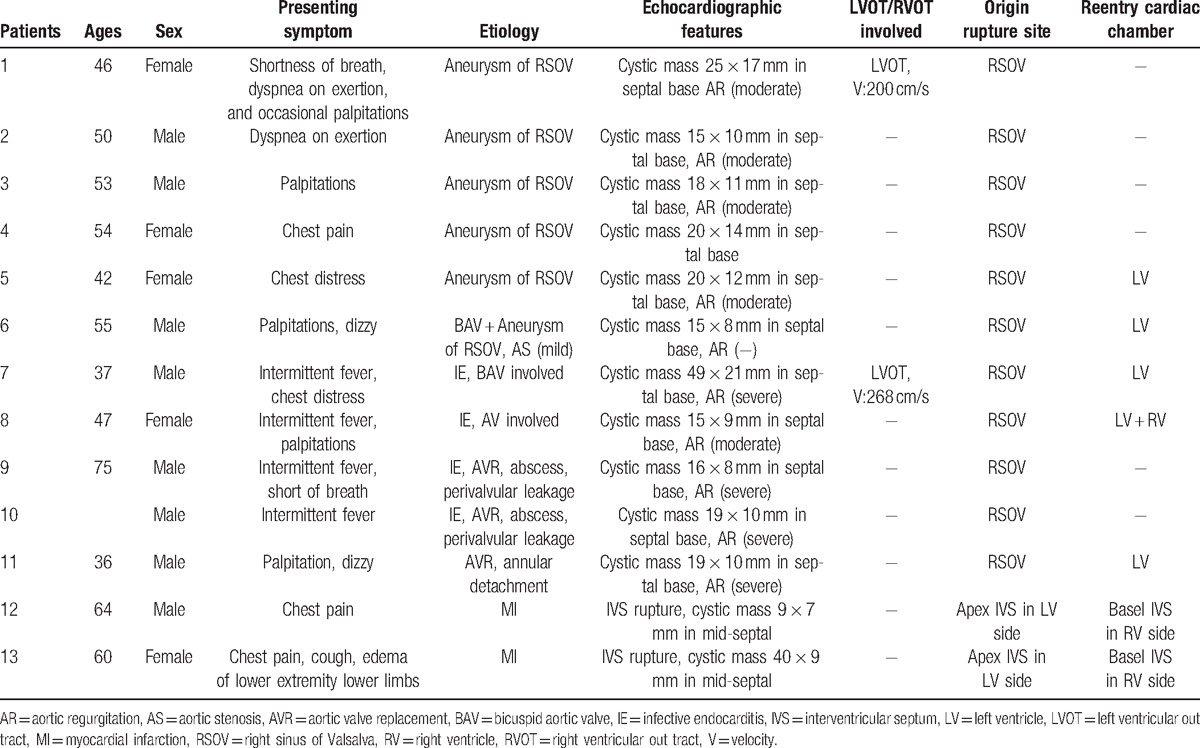
The clinical data, echocardiographic findings and pathological details of the 13 patients.

**Figure 1:**
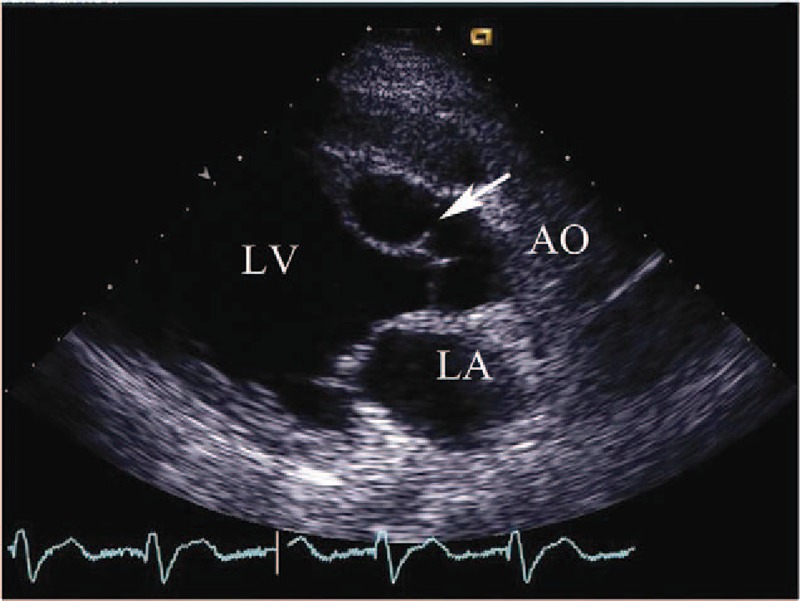
(Patient #2) Transthoracic echocardiogram (parasternal long axis view) showed the right sinus of Valsalva aneurysm and the entry site of rupture (arrow) into the dissecting cystic-like cavity in the interventricular septum. AO = aorta, LA = left atrium, LV = left ventricle.

**Figure 2:**
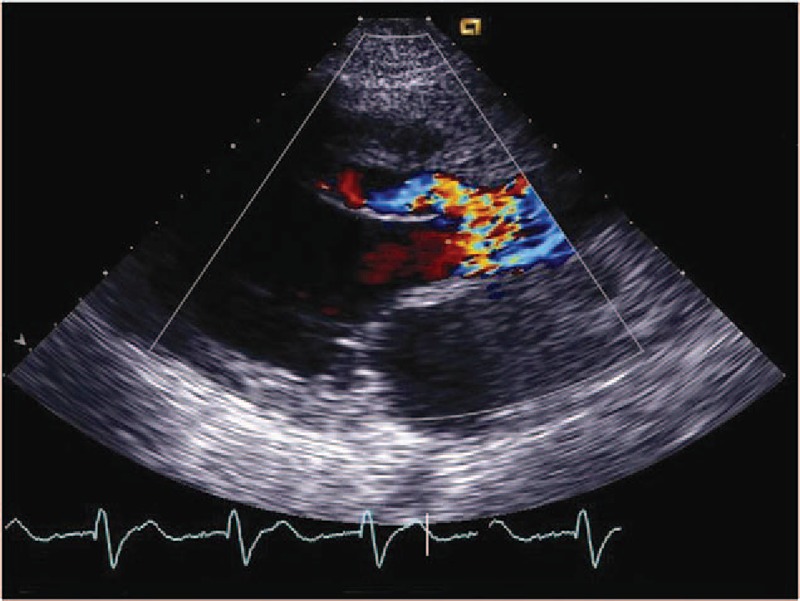
(Patient #2) Transthoracic echocardiogram (parasternal long axis view with color) showing the communication between the right sinus of Valsalva aneurysm and the dissection in the interventricular septum.

**Figure 3:**
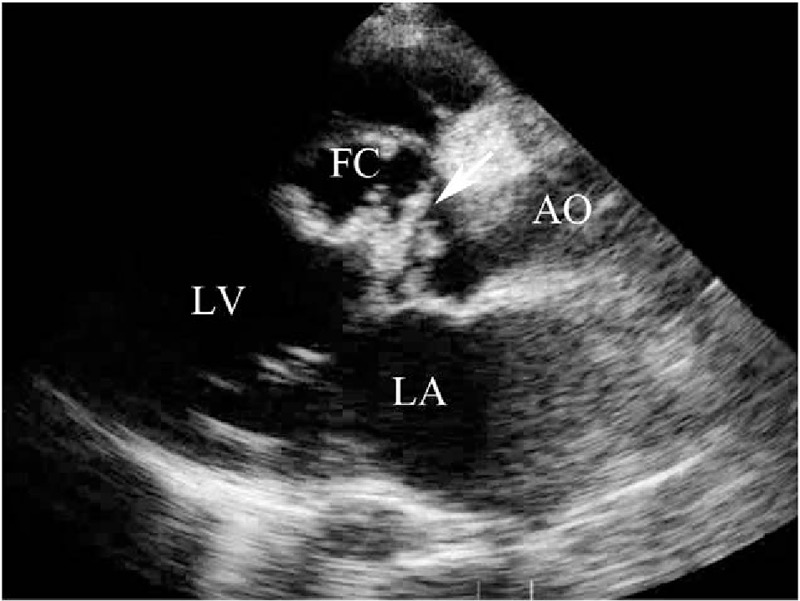
(Patient #7) Transthoracic echocardiogram (parasternal long axis view) showing thickened aortic valve leaflets with vegetations and the entry site of rupture (arrow) from the right sinus of Valsalva into the dissecting cystic-like false cavity in the interventricular septum (see also video 1). AO = aorta, FC = false cavity, LA = left atrium, LV = left ventricle.

**Figure 4:**
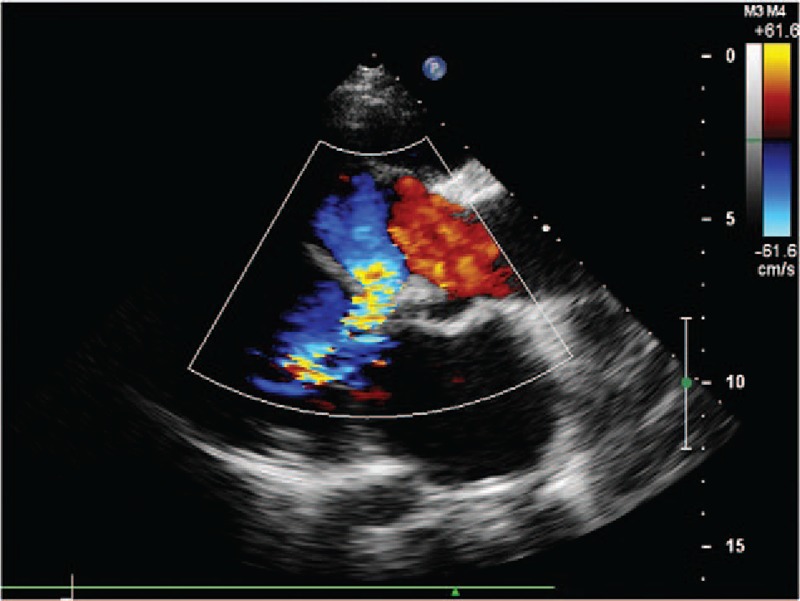
(Patient #7) Transthoracic echocardiogram (parasternal long axis view with color) showing both the communication between the right sinus of Valsalva aneurysm and the dissecting cavity in the interventricular septum and the communication between the dissecting cavity in the interventricular septum and the left ventricle (see also video 2).

**Figure 5:**
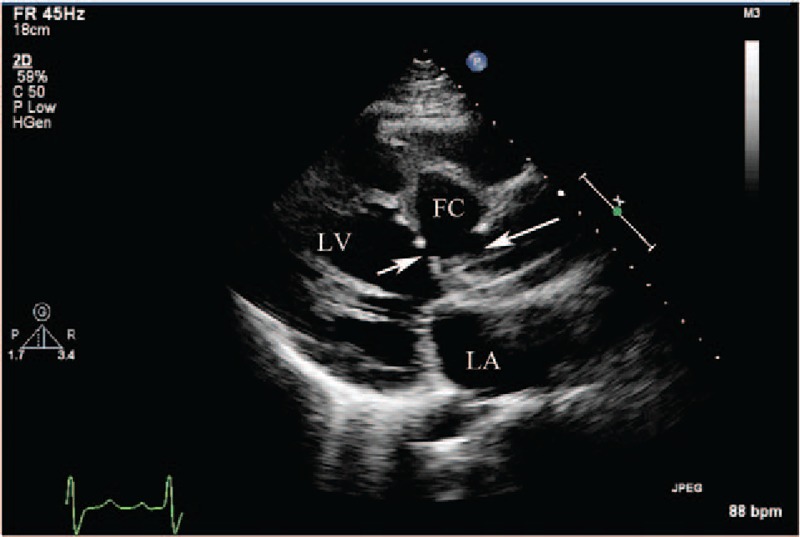
(Patient #11) Transthoracic echocardiogram (parasternal long axis view) showing a mechanical aortic valve in situ with annular detachment and the entry site of rupture (arrow on right) from the right sinus of Valsalva aneurysm into the dissecting false interventricular septal cavity, and the entry site of rupture (arrow on left) from the false interventricular septal dissecting cavity into the left ventricular cavity. (See also video 3). FC = false cavity, LA = left atrium, LV = left ventricle.

**Figure 6:**
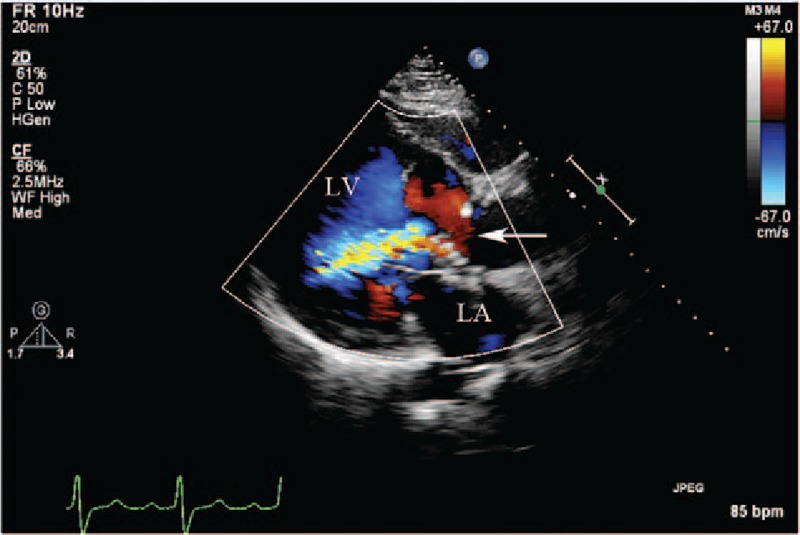
(Patient #11) Transthoracic echocardiogram (parasternal long axis view with color) showing the communication (arrow) from the right sinus of Valsalva aneurysm into the interventricular septal cavity, and the entry site of rupture from the false interventricular septal dissecting cavity into the left ventricular cavity. LA = left atrium, LV = left ventricle.

**Figure 7:**
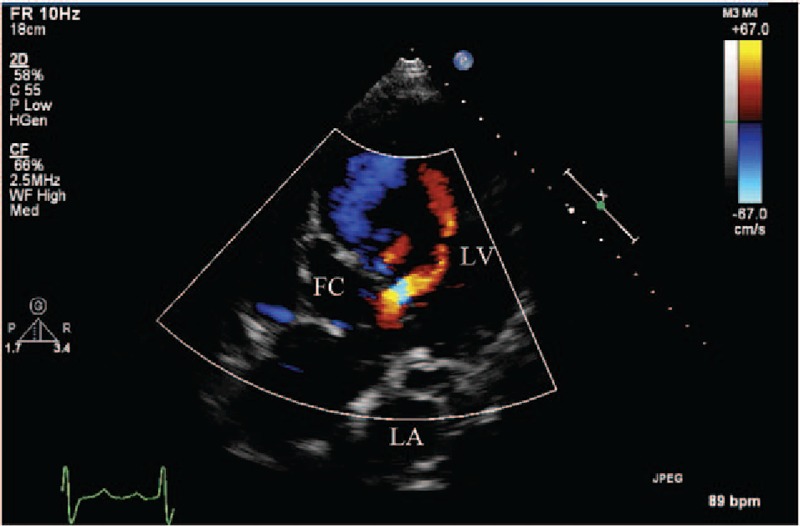
(Patient #11) Transthoracic echocardiogram (apical 5 chamber view with color) showing 2 flow jet communications from interventricular septal dissecting cavity to the left ventricular cavity. FC = false cavity, LA = left atrium, LV = left ventricle.

**Figure 8:**
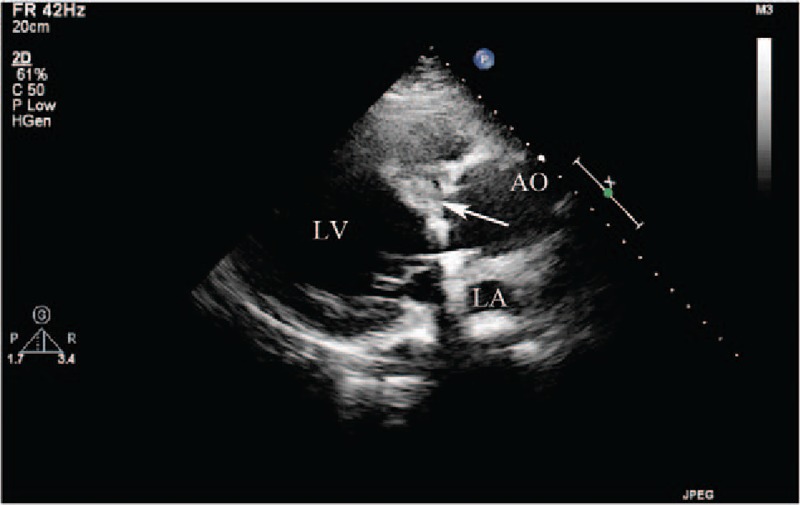
(Patient #11) Transthoracic echocardiogram (parasternal long axis view) showing a Dacron patch used to repair the rupture of right sinus of Valsalva (arrow) and to repair the annular detachment of the mechanical valve. AO = aorta, LA = left atrium, LV = left ventricle.

Transthoracic echocardiograms showed that interventricular septal dissection involved the right sinus of Valsalva (SOV) in 11 patients. In 5 of those patients, normal tricuspid aortic valves were seen (Figs. 1 and 2). Only 1 patient had a bicuspid aortic valve associated with a right SOV aneurysm. In 4 of the 11 patients, endocarditis involving the aortic valve was the likely cause of the interventricular dissection. Among these 4 patients, 1 patient had infection of the tricuspid aortic valve tissue, 1 patient had infection of the bicuspid valve tissue, and 2 patients had abscess formation adjacent to an aortic valve replacement (Figs. 3 and 4). In 1 patient who had undergone a mechanical aortic valve replacement and subsequent interventricular septal dissection, annular detachment of the aortic valve associated with a severe paravalvular leak was recorded (Figs. 5–8). In all 11 patients where the right SOV was involved, transthoracic echocardiograms showed the dissection as a cystic mass either confined to the base of the IVS or involving the base to the mid IVS. Furthermore, communication was observed between the interventricular dissection and the aortic root in all 11 patients.

With flow communicating from the aortic root to the interventricular cystic mass-like aneurysm, the cystic mass-like aneurysm pulsated with the cardiac cycle, increasing during diastole, and decreasing during systole. Two patients had increased left ventricular outflow tract (LVOT) flow with velocities of 268 and 200 cm/second, respectively. There were perforation sites noted between the IVS dissection aneurysm and the left ventricular cavity in 2 patients and a perforation site between the IVS dissection aneurysm and both left and right ventricular cavities in 1 patient. No perforation sites were noted in the other 8 patients.

**Figure 9:**
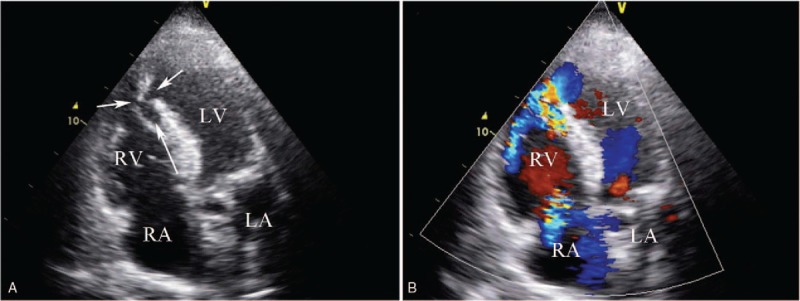
(Patient #12) Transthoracic echocardiogram (apical 4 chamber views) showing interventricular septal dissection following an anterior septal myocardial infarction. (A) The short arrows indicate the rupture of the interventricular septum both in left ventricle and right ventricle side. (B) Color flow (long arrow) shows the dissecting cavity of interventricular septum. LA = left atrium, LV = left ventricle, RA = right atrium, RV = right ventricle.

In 2 patients, IVS dissection followed septal rupture due to a myocardial infarction, resulting in perforation sites between the IVS dissection and the right ventricle (Fig. 9). The TTE showed irregular serpiginous tracts that appeared to originate from the infarct site and extended to the distal myocardium, eventually apparently rupturing into the right ventricle. In 1 patient, perforation of the IVS was at same level, but in another patient, the perforation of the IVS was at different levels on the left ventricular side versus the right ventricular side. The dissection also pulsated with the cardiac cycle, increasing during systole with flow communicating from the left ventricle through the IVS dissection, and decreasing during diastole.

## 4 Discussion

IVS dissection is a rare complication of an SOV aneurysm, infective endocarditis, cardiac surgery, or chest trauma. The complications have been featured in individual case reports. Few studies focus on the etiology and the findings of IVS dissection on TTE. A review of our database found 13 patients with IVS dissection. The TTE features of IVS dissection and its etiologies are summarized in this paper.

Right SOV aneurysm is the most prevalent etiology of IVS dissection. It is a rare anomaly of the aortic root, related to congenital defects of the aortic media and only rarely resulting from acquired conditions.^[7]^ The frequency of reported SOV aneurysms is 0.14% to 1.5%.^[8–10]^ Congenital SOV aneurysms are more common than acquired SOV aneurysms. They invariably rupture into the right ventricle and/or the right atrium resulting in an aortopulmonary shunt. In this study, 6 right SOV aneurysms were present in 13 cases of IVS dissection (46%).

Complications of SOV aneurysms include aortic insufficiency, coronary artery flow compromise, arrhythmias, and rupture. Rupture occurs most frequently from the right SOV into the right ventricle or right atrium. However, rupture from the SOV may also occur into the pericardium, the pleural space, or the left heart chambers.^[11]^ Rupture into the IVS is 1 of the more common complications of an SOV aneurysm, mostly originating from the right SOV. Ruptures of left or noncoronary SOV aneurysms into the IVS are extremely rare.^[12]^ Furthermore, the mechanisms of these aneurysmal ruptures into the IVS remain obscure. One possible explanation is that an SOV aneurysm compresses the coronary arteries leading to ventricular septal ischemia.^[13]^

It has been suggested that extension of an IVS dissection may be caused by intramural rupture of a congenital aneurysm with subsequent formation of a hematoma.^[14–19]^ This has been reported to possibly be a pseudoaneurysm, because no true aneurysmal wall has been found in these cases during autopsy studies. Wu et al^[6]^ have suggested that an SOV aneurysm is not the only cause of dissection of the IVS. In 3 of their 6 reported patients, the SOV were normal, and they concluded that intrinsic changes in the IVS should not be overlooked. They also suggested that embryological defects of the aortic annulus and IVS may contribute to the pathology.^[3,20]^

Infective endocarditis appears to be the 2nd common etiology of IVS dissection in this study. Four patients had infective endocarditis as a cause of their IVS dissection (31%), 1 of which had a bicuspid aortic valve, and 2 IVS dissections occurred after aortic valve replacement. Other etiologies of IVS dissection include aortic valve endocarditis with abscess formation through the IVS tissues.

IVS dissection developing after aortic valve replacement is even rarer, and considered a long-term complication of aortic valve replacement. It has a progressive course, carries a poor prognosis, and is associated with aortic valve insufficiency and annular dilatation.^[21]^ Aortic prosthetic annular detachment was seen in 1 patient in our study (patient 10). The mechanism for the detachment of the aortic valve prosthesis is thought to be postoperative shear stress due to turbulent flow between the IVS and the prosthetic valve.^[22]^

IVS dissection may also occur as a complication of acute myocardial infarction. This complication carries a high mortality rate, even after surgical repair.^[23]^ Mariscalco et al^[24]^ have reported IVS dissection postmyocardial infarction without rupture into the left or right ventricles or into the pericardial space. In our study, 2 patients that had IVS dissection complicating their acute myocardial infarction; both ruptured into the right ventricular cavity. The mechanism for the myocardial muscular fiber dissection is not clear, but it is suggested that the helical myocardial muscular band structure may influence the trajectory of the infiltrating hemorrhage between the muscular bands.^[25]^

TTE is a suitable imaging technique to diagnose IVS dissection. In addition to identifying the features of IVS dissection, it is also useful to detect the possible etiology. In 11 of the patients in our study, the dissected segment of the IVS from the base to mid-septum was visualized as a cystic mass-like aneurysm, and it was possible to track the aneurysm from its origin in the right SOV. In each of these 11 patients, the right coronary sinus was the site of origin. The pulsating cystic aneurysm appeared in various sizes depending on the cardiac cycle, increasing during diastole with flow communicating from aortic root to the aneurysm, and decreasing during systole.

The cystic mass-like aneurysm may or may not rupture into the left or right ventricular cavity, or into the pericardial space. Among our 11 patients, there was no shunt flow between the IVS dissection and a cardiac chamber or the pericardial cavity in 8 patients. In the remaining 3 patients, 2 ruptured into the left ventricle and 1 patient ruptured into both the left and right ventricles.

The presence of IVS dissection rupture into a ventricular cavity appears to depend on several factors, including the amount of blood flowing through the rupture, the speed with which the rupture develops, and the particular cardiac chamber into which the rupture occurs.^[26]^ Also, intermittent hemodynamic obstruction of the LVOT has been reported due to the pulsatile dissected IVS wall.^[27]^ In our study, 2 patients showed increased LVOT flow with Doppler flow velocities of 268 and 200 cm/second, respectively.

Two of our patients had IVS dissection following acute myocardial infarction. The echocardiographic characteristics of the dissecting cystic mass-like aneurysms were seen in the IVS. In 1 patient, the septal perforations were at the same level into both the left and right ventricles. In the other patient, the rupture was only into the right ventricle and at a different area of the IVS.

Although the cystic mass-like aneurysm of IVS dissection is seen to pulsate with each cardiac cycle, the pulsation changes according to its etiology and location. In dissection rising from the right SOV, the cystic mass-like aneurysm increases during diastole with flow communicating from the aortic root to the interventricular cystic mass-like aneurysm, due to the higher pressure in the aorta compared to left ventricular diastolic pressure. Conversely, in myocardial infarction patients, the cystic mass-like aneurysm increases during systole with flow communicating from the left ventricle to the IVS dissection, because in systole, the pressure in the left ventricle is higher.

The presenting clinical features of IVS dissection vary and may include palpitations, syncope, and dyspnea. Other reported clinical manifestations include congestive heart failure, arrhythmia, and heart block. Treatment is advocated for symptomatic patients and for asymptomatic patients at risk for any possible complications. Possible management options for IVS dissection include surgical correction or transcatheter closure.^[26]^ The objectives of surgical management of IVS dissection include restoration of the integrity of the ventricular septum, resection of the SOV aneurysm, and obliteration of any obstruction of the left and right ventricular outflow tracts, thereby improving cardiac function.^[6]^

## 5 Limitations

The principal limitations of this study are its retrospective construction in a single center with a relatively small number of patients. A larger number of patients with systemic analysis are needed.

## 6 Conclusions

This study has shown that IVS dissection usually commences in the right SOV, due to either SOV aneurysm or infective endocarditis, following aortic valve replacement or as a complication of myocardial infarction. The transthoracic echocardiographic characteristic of IVS dissection is a cystic-like mass seen in the IVS.
